# C-reactive protein and albumin kinetics before community-acquired bloodstream infections – a Danish population-based cohort study

**DOI:** 10.1017/S0950268820000291

**Published:** 2020-02-26

**Authors:** O. S. Garvik, P. Póvoa, B. Magnussen, P. J. Vinholt, C. Pedersen, T. G. Jensen, H. J. Kolmos, A. T. Lassen, K. O. Gradel

**Affiliations:** 1 Research Unit of Clinical Epidemiology, Institute of Clinical Research, University of Southern Denmark, and Center for Clinical Epidemiology, Odense University Hospital, Kløvervænget 30, Entrance 216, ground floor, 5000 Odense C, Denmark; 2 NOVA Medical School, New University of Lisbon, Campo Mártires da Pátria 130, 1169-056 Lisbon, Portugal; 3 Polyvalent Intensive Care Unit, São Francisco Xavier Hospital, CHLO, Estrada do Forte do Alto do Duque, 1449-005 Lisbon, Portugal; 4 Department of Clinical Biochemistry and Pharmacology, Odense University Hospital, Sdr. Boulevard 29, entrance 40, 5000 Odense C, Denmark; 5 Department of Infectious Diseases, Odense University Hospital, Sdr. Boulevard 29, entrance 20, 5000 Odense C, Denmark; 6 Department of Clinical Microbiology, Odense University Hospital, J.B. Winsløws Vej 21, 2nd floor, 5000 Odense C, Denmark; 7 Department of Emergency Medicine, Odense University Hospital, Kløvervænget 25, entrance 63-65, 5000 Odense C, Denmark

**Keywords:** Albumin, C-reactive protein, community acquired bloodstream infections

## Abstract

Early changes in biomarker levels probably occur before bloodstream infection (BSI) is diagnosed. However, this issue has not been fully addressed. We aimed at evaluating the kinetics of C-reactive protein (CRP) and plasma albumin (PA) in the 30 days before community-acquired (CA) BSI diagnosis. From a population-based BSI database we identified 658 patients with at least one measurement of CRP or PA from day −30 (D–30) through day −1 (D–1) before the day of CA-BSI (D0) and a measurement of the same biomarker at D0 or D1. Amongst these, 502 had both CRP and PA measurements which fitted these criteria. CRP and PA concentrations began to change inversely some days before CA-BSI diagnosis, CRP increasing by day −3.1 and PA decreasing by day −1.3. From D–30 to D–4, CRP kinetics (expressed as slopes – rate of concentration change per day) was −1.5 mg/l/day. From D–3 to D1, the CRP slope increased to 36.3 mg/l/day. For albumin, the slope between D–30 to D–2 was 0.1 g/l/day and changed to −1.8 g/l/day between D–1 and D1. We showed that biomarker levels begin to change some days before the CA-BSI diagnosis, CRP 3.1 days and PA 1.3 days before.

## Introduction

Early changes in biomarkers probably occur some days before bloodstream infection (BSI) is diagnosed. This issue has not been fully addressed in previous clinically based studies, in particular in community-acquired (CA) infections [1–4]. The assessment of biomarker kinetics before BSI diagnosis could provide knowledge on the basic understanding of underlying mechanisms of infection as well as improving the usefulness of biomarker kinetics in the clinical decision-making process.

Hospital-acquired infections frequently occur in patients with other non-infectious conditions that are associated with increased biomarker levels, e.g. previous surgery or IV lines. As CA infections occur in a previously healthier population, without those major inflammatory stimuli, they make a better model for studying biomarker kinetics before infection diagnosis.

An additional problem is the definite diagnosis of infection. Frequently, we are unable to obtain the microbiological documentation of an infection. This is particularly true in CA infections, e.g. due to previous antibiotics prescriptions and difficulty in obtaining good quality samples for microbiology, in particular in lower respiratory tract infections in patients without tracheal intubation. However, CA-BSI is a well-defined clinical entity based on clear and globally accepted clinical and microbiological diagnostic criteria [5].

The Danish Observational Registry of Infectious Syndromes (DORIS) is a population-based research database comprising CA-BSI patients as well as their data before the reference BSI episode, namely biochemistry and microbiological data [6].

Consequently, the DORIS database enabled us to overcome the above-mentioned limitations of a CA infection definite diagnosis. In the present study, we aimed at evaluating the kinetics of C-reactive protein (CRP) and plasma albumin (PA) in the 30 days before the CA-BSI episode.

## Methods

### Setting

The Danish national health system is tax financed, covering both primary and hospital care. All residents have a unique civil registration number used for all health contacts and linkages between health administrative registries [7]. The admission of all residents with acute illnesses from a well-defined geographical region to a hospital prompted a population-based study based on data from registries and the medical records in the DORIS research database [6]. The study was approved by the Danish Data Protection Agency (record no. 2013-41-2579). Approval by an ethics committee or consent from participants is not required for registry-based research in Denmark.

### Initial study cohort

The study cohort has been described in detail elsewhere [6]. In brief, it comprised all adults (>14 year) residing in the Funen County with the first episode of CA-BSI, in the period 2000–2008, a total of *N* = 2785 patients.

A CA-BSI was defined as BSI occurring <3 days after hospital admission and without inpatient contact in the preceding seven days. We had all the CA-BSI patients' hospital recorded CRP and PA measurements from 2000 through 2008 [8]. Unrealistically low levels of PA (<11 g/l) were excluded. The CRP values below the limit of detection (<10 mg/l, *N* = 452 (14.5%)) were randomly allocated to a value in the range 0–9 mg/l, as described elsewhere [8]. From this cohort we have derived different sub-cohorts based on the occurrence of CRP and/or PA within pre-specified time periods around the date of CA-BSI [8–10].

### Final study cohort

We included CA-BSI patients with at least one measurement of CRP or PA from day −30 (D–30) through day −1 (D–1) before the day of CA-BSI (D0) and a measurement of the same biomarker at D0 or D1. D0 is defined as the day the blood culture is drawn.

### Analyses of CRP and PA levels

All analyses were performed by the Department of Clinical Biochemistry and Pharmacology, Odense University Hospital, with the results recorded in Netlab (Medasys S.A., Littau, Switzerland). CRP was measured by an immunoturbidimetry method on Modular P^®^ (Roche, Mannheim, Germany). PA was also measured on Modular P^®^ by the use of a bromocresol green dye-binding method. All specimen dates refer to the date of drawing blood specimens.

### Statistical analysis

CRP levels were not normally distributed whereas PA levels were. Because the results for means and medians were materially the same, we report the mean levels. CRP and PA concentrations were found to change their course prior to D0. This behaviour was modelled as a broken stick where two linear functions were defined on two distinct time-intervals, (D–30,*c*) and(*c*,  D1) [11]. The intersection of the functions defined *c* as the point in time in which the concentration course changed and the parameters were determined by a broken stick regression (BSR). Differences in the dynamics of monomicrobial Gram-positive, monomicrobial Gram-negative and polymicrobial CA-BSIs were investigated by estimating distributions of the parameter-differences by a bootstrap algorithm (1000 replications) [12]. A distribution with a 95%-confidence interval (CI) in which zero was not included would imply significance for the parameter-difference.

We used the program Stata, *vs.* 14 (StataCorp, College Station, TX, USA) for all analyses.

## Results

Among 658 patients, 576 fulfilled the criteria of CRP on D–30/−1 and D0/D1, 584 fulfilled this for PA, whereas 502 patients fulfilled this for both CRP and PA. The baseline clinical characteristics at the day of CA-BSI diagnosis are presented in Table 1. The microbiological isolates are shown in Table 2.

**Table 1.** Baseline patient characteristics (*n* = 658)

	Number (%)^a^
Measurements of CRP and PA	
Only CRP measured	74 (11.3)
Both CRP and PA measured	502 (76.3)
Only PA measured	82 (12.5)
Age, years	
Mean, s.d.	66.1, 15.6
Females	279 (42.4)
Charlson comorbidity index	
0 points	42 (6.4)
1–2 points	278 (42.3)
>2 points	338 (51.4)
Antibiotics redeemed from pharmacy	
No redemption 0–30 D before CA-BSI	534 (81.2)
Redemption 8–30 D before CA-BSI	86 (13.1)
Redemption <8 D before CA-BSI	38 (5.8)
Admitted to	
Surgical ward	58 (8.8)
Medicine ward	419 (63.7)
Oncology/haematology ward	149 (22.6)
Intensive care unit	32 (4.9)
Sepsis severity	
No sepsis	49 (7.5)
Possibly sepsis	120 (18.2)
Sepsis	156 (23.7)
Severe sepsis or septic shock	293 (44.5)
Organ dysfunction without sepsis	40 (6.1)
Number of organ failures	
0	325 (49.4)
1	222 (33.7)
⩾2	111 (16.9)
30-Day mortality	161 (24.5)

CRP, C-reactive protein; PA, plasma albumin; s.d. standard deviation.

a Except for ‘Age, years’, cf. text.

**Table 2.** Microbiological isolates (*n* = 658)

Text	Number (%)
Monomicrobial Gram-positive	224 (34.0)
*Staphylococcus aureus*	116 (17.6)
*Streptococcus pneumoniae*	52 (7.9)
*Streptococci*, other	28 (4.3)
*Enterococcus faecalis*	28 (4.3)
Monomicrobial Gram-negative	353 (53.6)
*Escherichia coli*	202 (30.7)
*Klebsiella* spp.	48 (7.3)
*Pseudomonas aeruginosa*	35 (5.3)
Other	68 (10.3)
Polymicrobial	81 (12.3)

### CRP kinetics before CA-BSI diagnosis

**Fig. 1.**
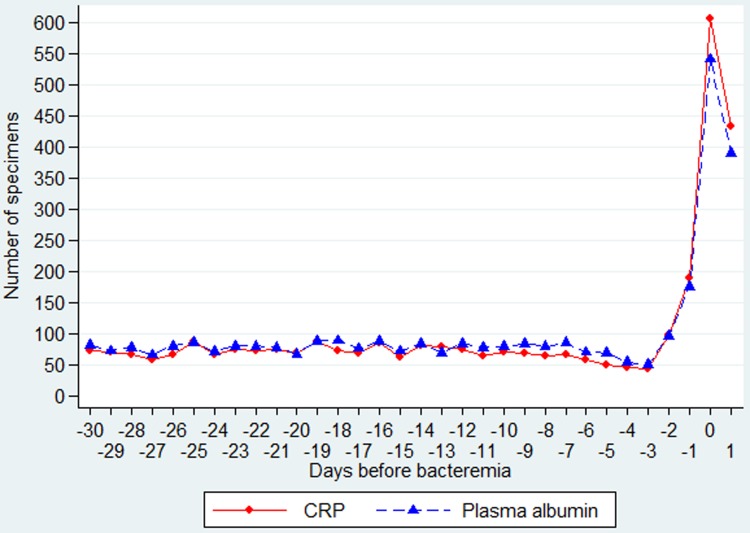
Daily number of specimens of CRP and PA from day −30 through day 1 (in relation to the day of the CA-BSI).

**Fig. 2.**
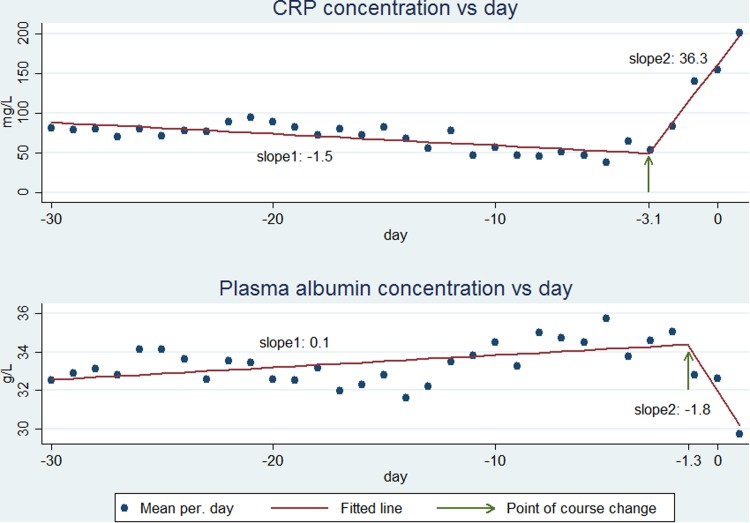
CRP (upper panel) and PA (lower panel) course from day −30 through the day after CA-BSI. The observed mean values (points) are fitted as two linear functions (lines) which are connected at the point of course change (indicated by an arrow).

Among the 576 patients, the daily number of measurements was fairly constant until D–3 (range 44–87), increasing to 98 on D–2, 189 on D–1, 607 on D0, with a decline to 434 on D1 (Fig. 1). The CRP values were already elevated (>60 mg/l) in most of the 30 days before CA-BSI (Fig. 2, upper panel).

CRP began to increase some days before D0 (Fig. 2, upper panel). The BSR analysis showed that CRP began to increase at day −3.1 (95% CI−3.6 to −2.6) (Fig. 2, upper panel, arrow). The day of CRP change did not differ significantly between monomicrobial Gram-positive (day −2.8, 95% CI −3.7 to −1.9), monomicrobial Gram-negative (day −3.1, 95% CI −3.8 to −2.4) or polymicrobial CA-BSI (day −3.4, 95% CI −4.6 to −2.2) (data not shown). From D–30 to D–4, the CRP kinetics, expressed as a slope (rate of concentration change per day), was −1.5 mg/l/day (95% CI −2.0 to −0.9), whereas the slope was 36.3 mg/l/day (95% CI 31.3–41.4) from D–3 to D1 (Fig. 2, upper panel, slope 1 and slope 2, respectively). In the two time periods, the slopes for monomicrobial Gram-positive, monomicrobial Gram-negative or polymicrobial CA-BSIs were not significantly different (−1.3 (95% CI −2.3 to −0.2), −1.3 (95% CI −2.0 to −0.5) and −2.7 (95% CI −4.2 to −1.2) mg/l/day; 39.7(95% CI 28.6–50.7), 34.0 (95% CI 27.2–40.9) and 40.5 (95% CI 27.3–53.6) mg/l/day).

**Fig. 3.**
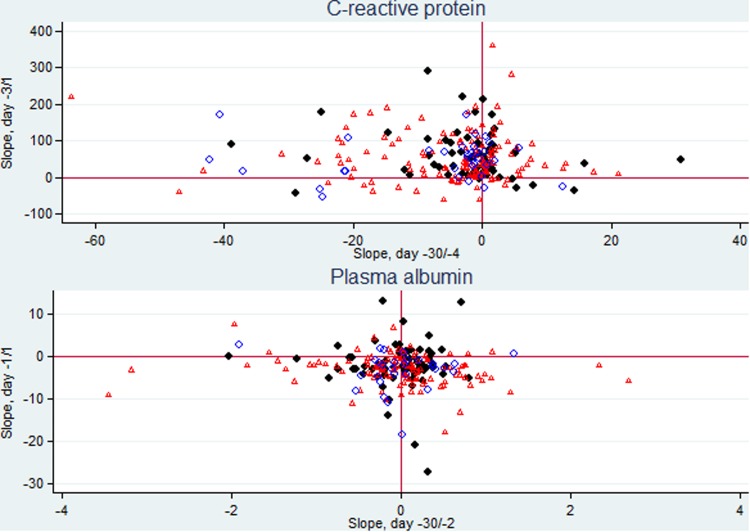
Scatter plots of CRP individual slopes from day −30 (D–30) through day −4 and from day −3 through the day after CA BSI (D1) (226 patients) and PA individual slopes from D–30 through day −2 and from day −1 through D1 (210 patients). Patients with monomicrobial Gram-positive CA-BSI are presented as dots, with monomicrobial Gram-negative CA-BSI as triangles, and with polymicrobial CA-BSI as circles.

Focusing on the 226 patients having ⩾2 CRP specimens in both periods (data not shown) we assessed their individual CRP kinetics (slopes), from D–30 to D–4 and from D–3 to D1. Most of the patients presented almost no variation or a slight negative slope in the first period followed by a marked increase in the days just before CA-BSI diagnosis in 84.5% of the patients (Fig. 3, upper panel).

### PA kinetics before CA-BSI diagnosis

Among the 584 patients, the daily number of measurements was fairly constant until D–3 (range 50–89), increasing to 96 on D–2, 176 on D–1, 542 on D0, with a decline to 390 on D1 (Fig. 1).

The PA values were low (<35 g/l, lower limit of the reference range) in most of the 30 days before CA-BSI (Fig. 2, lower panel).

PA began to decrease before D0 (Fig. 2, lower part). The BSR analysis showed that PA began to decrease at day −1.3 (95% CI −2.4 to −0.8) (Fig. 2, lower panel, arrow). For monomicrobial Gram-positive CA-BSIs, the day of change was −0.3 (95% CI−0.8 to 0.2) which was not significantly different from D0. The day of change for monomicrobial Gram-negative −1.1 (95% CI −1.7 to −0.6) and polymicrobial −6.4 (95% CI −11.3 to −1.48) CA-BSIs differed significantly from D0. The days of change for monomicrobial Gram-positive and monomicrobial Gram-negative CA-BSIs were not significantly different, whereas numbers for polymicrobial CA-BSIs were too low for robust computations.

From D–30 to D–2 PA kinetics, expressed as a slope, were 0.1 (95% CI 0.03–0.10) g/l/day whereas from D–1 to D1 the slope changed to −1.8 (95% CI −2.4 to −1.3) g/l/day (Fig. 2, lower panel, slope 1 and slope 2, respectively). In the first time period, the slopes of monomicrobial Gram-positive (−0.003 g/l/day, 95% CI −0.05 to 0.05) and polymicrobial CA-BSIs (0.11 g/l/day, 95% CI −0.01 to 0.22) were not significantly different from zero. The slope of the monomicrobial Gram-negative CA-BSI was 0.09 g/l/day (95% CI 0.05–0.14). In the second time period, the slopes of monomicrobial Gram-positive −2.9 g/l/day (95% CI −4.3 to −1.5) and monomicrobial Gram-negative −2.3 g/l/day (95% CI −3.0 to −1.6) were not significantly different. The slope of the polymicrobial CA-BSI was −0.6 g/l/day (95% CI −1.1 to −0.1).

Focusing on the 210 patients having ⩾2 PA specimens in both periods (data not shown) we assessed the individual PA kinetics (slopes), from D–30 to D–2 and from D–1 to D1. We found that most of the patients presented almost no variation or a minor positive slope during the first period followed by a negative slope in the days before CA-BSI diagnosis in 78.6% of the patients (Fig. 3, lower panel).

## Discussion

We found that CRP and PA concentrations began to change inversely some days before diagnosing CA-BSI, CRP increasing by day −3.1 and PA decreasing by day −1.3. The assessment of CRP kinetics clearly showed that before D–3, CRP presented a slight negative slope followed by a sharp and marked increase in the days before CA-BSI diagnosis. PA presented an inverse course in comparison with CRP with a very slight positive slope before D–1 followed by a decrease.

As already pointed out by our group [8], CRP and PA levels were not within the normal range before the CA-BSI. The elevated CRP concentration, >60 mg/l, is much higher than the <10 mg/l found in healthy Danes [13, 14]. The same was true for PA, showing a slight hypoalbuminemia during the study period. High CRP and low PA in patients in whom the physician found an indication to perform blood tests could be a surrogate marker of an underlying condition associated with some levels of chronic inflammation before the development of CA-BSI.

The mechanisms of the host response to infection have been well studied in animal models but such data are difficult to extrapolate to clinical reality. In daily life, patients frequently go to their general practitioner or an emergency department with unspecific clinical complaints which have often lasted for days. These unspecific conditions may later develop into a full-blown clinical picture caused by a well-established infection. As a result, these patients do not present to the emergency departments within a couple of hours of disease course, as is the case of e.g. acute myocardial infarction [15].

It is generally difficult to define the beginning of infectious diseases. The DORIS database enabled us to explore the kinetics of CRP and PA before the clinical diagnosis of CA-BSI. Our study clearly showed that in these CA-BSI patients, with blood tests performed in the preceding 30 days, CRP began to increase 3.1 days before the diagnosis and PA began to decline 1.3 days before the diagnosis. Before these two time-points, both biomarkers remained fairly unchanged, which was reflected by the very low slopes. But afterwards, maybe reflecting the initiation of the infectious process and the associated host response, we noticed marked changes in kinetics with a sharp increase in the CRP slope and a decrease in the PA slope. This indicates that the infection is a dynamic process that begins to elicit an inflammatory response before the clinical diagnosis. One could consider that these unspecific clinical manifestations were not severe enough to make the patient seek medical consultation, or, if they did, the symptoms were not severe enough to indicate taking blood cultures or hospital admission. But the initial host response to the infection was already signalled by inverse changes in the CRP and PA slopes.

We could speculate that in patients having a high risk of infectious complications, CRP and PA kinetics, in addition to symptoms/signs (such as fever or tachycardia) suggestive of an infection, could prompt the physician to order further tests (e.g. blood cultures) and possibly prescribe pre-emptive antibiotic therapy [16]. Our results also point to the usefulness of the combined use of CRP and PA.

Our study has several strengths. To our knowledge, apart from two studies in the same cohort [8, 10], no study has assessed longitudinal measurements of CRP and PA before a CA infection. Secondly, it comprises a large cohort of CA-BSI episodes, which increases the robustness and consistency of our findings. Thirdly, BSIs are infections defined according to well-defined and globally accepted criteria [17]. Finally, our cohort derives from a population-based database.

Our study also has several limitations. Firstly, it is retrospective although the data *per se* were prospectively collected from registries and medical records. Secondly, the number of specimen dates per patient before D0 varied (from 1 to 26) and the measurement of CRP/PA depended on the physician's assessment and the patient's clinical complaints. However, our study's main focus was patterns related to longitudinal data. A recent study comprising 2418 of the initial larger cohort of 2785 patients [6] showed that these patterns were materially the same regardless of the numbers of specimens [10], which facilitates the extrapolation of results between patient groups having different numbers of specimens in the different time periods around CA-BSI. Thirdly, we had not enough clinical data to interpret whether the abnormal CRP and PA levels before the day of slope change could reflect an underlying disease causing some degree of chronic inflammation. We speculate that CA-BSI patients with no blood tests before D0 would be healthier, and consequently would have lower CRP and higher PA. Our group has also found that this latter group had 50% lower 30-day mortality [10]. Fourthly, the D0 was influenced by two variables that cannot be controlled in studies on CA-BSI, one is the patient's own decision to attend an emergency department and the other is the physician's own decision to collect blood cultures. Fifthly, we had no data on nutrition. However, for the larger CA-BSI cohort we found a high negative correlation (*R* = −0.47, *P* < 0.0001) between 61 583 paired CRP and PA specimens, clearly indicating hypoalbuminemia as a marker of an underlying chronic inflammation [8]. This is also in accordance with several reviews [18, 19]. Sixthly, our definition of CA differed from the more generally accepted definition of healthcare-associated infection [20] as many of our patients had a hospital contact in the 30 days before their CA-BSI episode [21]. However, this probably has little impact on biomarker kinetics before CA-BSI [10].

In conclusion, biomarker levels began to change some days before the CA-BSI diagnosis. For these patients, a sudden increase in the CRP followed shortly thereafter by a PA decrease could signal a high risk of CA-BSI.
